# An Observational Registry of Carotid Endarterectomy and Carotid Artery Stenting in Brazil: Study Protocol

**DOI:** 10.2196/resprot.5986

**Published:** 2016-11-23

**Authors:** Edwaldo Edner Joviliano, Winston Bonetti Yoshida, Marcone Lima Sobreira, Regina Moura, Ana Terezinha Guillaumon, Selma Regina De Oliveira Raymundo, Daniel Gustavo Miquelin, Ludvig Hafner, Marcelo Jose Almeida

**Affiliations:** ^1^ Department of Surgery and Anatomy Ribeirao Preto Medical School University of São Paulo Ribeirao Preto Brazil; ^2^ Department of Surgery Botucatu Medical School University Julio de Mesquita Filho UNESP Botucatu Brazil; ^3^ Department of Surgery School of Medical Sciences University of Campinas Campinas Brazil; ^4^ Department of Surgery São Jose do Rio Preto Medical School FAMERP Sao Jose do Rio Preto Brazil; ^5^ Department of Surgery Marilia Medical School FAMEMA Marilia Brazil

**Keywords:** carotid artery diseases, carotid endarterectomy, carotid stenosis, carotid stenting, medical record linkage

## Abstract

**Background:**

Carotid artery stenting (CAS) and carotid endarterectomy (CEA) are alternative strategies for stroke prevention in patients with atherosclerotic carotid disease. CEA has been considered the first-line treatment for carotid stenosis worldwide, and the safety and efficacy of CAS compared to CEA remains in question.

**Objective:**

The purpose of this study is to compare the practice and outcomes of CAS and CEA in a real-world setting within public university hospitals in Brazil.

**Methods:**

This study will be a prospective 5-year analysis of treatment for atherosclerotic carotid stenosis with CEA and CAS performed at 5 centers affiliated with the Vascular Study Group at public university hospitals in Brazil. The indications for the procedures will be determined by each surgeon’s individual discretion, in accordance with preoperative risk evaluation. The primary outcome measures will be (1) any in-hospital stroke or death, and (2) any per-procedural stroke, death, or myocardial infarction (MI). Patients undergoing CEA in conjunction with cardiac surgery will be excluded from the study. Multivariate logistic regression will be performed to identify predictors of stroke or death in patients undergoing CEA and CAS. All tests of significance will be performed at the .05 level. This study was approved by the Committee of Ethics in Research at the University Hospital of Ribeirao Preto Medical School, and in all other participating institutions linked to National Research System and National Board of Health in Brazil (Process 15695/2011).

**Results:**

This study is currently in the recruitment phase, and the final patient is expected to be enrolled by the end of 2018. We hope to recruit approximately 800 patients to the study. Analyses will focus on primary end points for patients that are allocated to each treatment group. During the per-procedural period, the occurrence of the primary end point components (stroke, MI, or death) for CAS and CEA will be analyzed for symptomatic or asymptomatic subjects.

**Conclusions:**

The analyses of the primary endpoints (and all others variables of the study) are expected to be published in 2019 in a peer reviewed journal, and results will be presented at scientific meetings, with summary results published online. This study will obtain new data related to the quality of treatment for carotid disease in Brazil at the primary training centers of future vascular surgeons, but the initial data that will be obtained and published (with the outcomes and complications) are restricted to the first 30 days postprocedure. This time restriction limits the comparison of the results that relate to the main goal of treatment, which is to decrease the risk of stroke over 5 years. The purpose of the study group is to continue the monitoring of patient records, and evaluate the follow-up data in the 5 years following the initial evaluation. This study protocol will contribute very significantly to improving the care of patients with carotid disease, in addition to qualifying the level of assistance provided in public university hospitals in the state of São Paulo, Brazil.

**Trial Registration:**

Clinicaltrials.gov NCT02538276; https://www.clinicaltrials.gov/ct2/show/NCT02538276 (Archived by WebCite at http://www.webcitation.org/6m7APnFLD)

## Introduction

Cerebrovascular disease is a leading cause of serious long-term disability and death [1,2]. A significant proportion of ischemic strokes originate from the atherosclerosis of extracranial arteries. In most cases, carotid endarterectomy (CEA) or stent angioplasty of the carotid bifurcation is considered when color duplex ultrasound detects clinically significant extracranial internal carotid artery stenosis. However, several clinical situations exist in which other imaging techniques are needed to provide greater anatomical detail and resolution.

Revascularization of severely atherosclerotic carotid arteries has been shown to be safe and effective in the prevention of stroke [2-5]. Carotid artery stenting (CAS) and CEA are two alternative methods of revascularization, and these techniques have been compared in small randomized clinical trials [6-10]. In the 2010 International Carotid Stenting Study (ICSS), 1713 symptomatic patients from 50 centers in Europe, Australia, New Zealand, and Canada were prospectively randomized to a CAS or CEA condition. The 30-day results showed a combined stroke, death, and myocardial infarction (MI) rate of 7.4% for CAS and 4.0% for CEA (*P*<.006). This effect was primarily driven by an increased stroke rate of 7.0% for CAS versus 3.3% for CEA [1].

Although randomized trials provide the most scientifically valid comparisons between treatments, they do not reflect the diversity of patients or technical proficiency present in broad contemporary clinical practice. CEA has been considered the first-line treatment for carotid stenosis worldwide, and the safety and efficacy of CAS compared to CEA remains in question. CAS is officially approved for use in multiple countries, and the number of patients undergoing CAS has been increasing due to its less-invasive nature. The present study aims to demonstrate the real-world status of the treatment of carotid artery disease with CAS and CEA using the prospective registry of vascular diseases of university hospitals in the state of Sao Paulo, Brazil, called the RHEUNI (Registry Project of Vascular Disease in the Public University Hospitals of São Paulo). It is well known that no prospective studies with a significant number of cases concerning the treatment of carotid disease exist in Brazil; all available data were derived from studies conducted in other countries. Thus, the main objective of this study is to evaluate the quality of treatment for carotid disease in Brazil by analyzing the two main techniques currently available. The data will be derived from the vascular surgery centers that bear primary responsibility for the training of future vascular surgeons.

## Methods

### Patient Population and Setting

The CEA and CAS registry in Brazil (part of RHEUNI) is being used as a prospective observational study in Brazil, and has been conducted since July 2013. Final data analyses are expected in July 2018 from the 5 public university hospitals of the São Paulo state that provide vascular therapy. Several consecutive procedures will be registered as CEA or CAS by certified vascular surgeons from 5 centers (University Hospital of Ribeirão Preto Medical School of University of São Paulo, University Hospital of School of Medical Sciences of University of Campinas, University Hospital of Marilia Medical School, University Hospital of Botucatu Medical School, and University Hospital of São Jose do Rio Preto Medical School). All participating vascular surgical centers have extensive experience in the treatment of carotid disease, and they are all training centers of reference in Brazil. The ethical committee of each hospital has approved this project.

Inclusion criteria are as follows: patients with carotid atherosclerotic stenosis >70% who underwent CEA or CAS for the treatment of carotid stenosis at any of the 5 hospitals involved in the study, male or female, and >18 years of age. Carotid stenosis is defined as (1) stenosis >70% by catheter angiography (North American Symptomatic Carotid Endarterectomy Trial criteria) or (2) by Doppler ultrasound with >70% stenosis defined by a peak systolic velocity of at least 230 cm/second, plus at least one of the following: an end diastolic velocity >100 cm/second, or internal carotid/common carotid artery peak systolic velocity ratio >4.0, or computer tomography with >70% stenosis, or magnetic resonance with >70% stenosis. Exclusion criteria include cases of concomitant cardiac surgery, carotid dissection, fibromuscular dysplasia, or trauma.

RHEUNI data have been validated for completeness by quarterly audits of discharge claims data from each participating institution. These audits ensure complete inclusion of all consecutive procedures performed at the participating hospitals.

### Data Collection

The recorded characteristics and backgrounds of patients who undergo CAS and CEA will include: age, gender, and high-risk CEA characteristics (according to the Stenting and Angioplasty with Protection in Patients at High Risk for Endarterectomy trial [6]). In addition, symptom presentation and the degree of stenosis will be analyzed. Procedural success, antiplatelet use, embolic protection device (EPD) use, and the type of stent strut (open-cell or closed-cell) or patch used for CEA will be recorded, and the execution of prior post balloon dilatation at CAS (and procedure-related complications) will be analyzed to clarify the current strategy and the treatment results of both techniques. The degrees of stenosis will be measured using the method employed by the North American Symptomatic Carotid Endarterectomy Trial [2].

### Data Source and Measurement

Outcomes will be stratified by symptomatic and asymptomatic status. Symptomatic patients will be defined as having a neurological event, including any hemispheric or ocular transient ischemic attack, or major or minor stroke, that precedes the intervention ipsilateral to the treated lesion. Our definition of a symptomatic patient is the occurrence of symptoms for up to 180 days. This definition is similar to that of the Carotid Revascularization Stent Trial (CREST), although ICSS trial lesions were considered symptomatic for up to one year [1,11,12].

Technical success pertains to per-procedural events from the initiation of the procedure through the first 24-hour post operative period. Primary technical success will require the successful excision of the carotid plaque by surgical or interventional means. Technical success will be assessed by the outcomes and complications related to preoperative carotid angiograms, whenever these imaging studies are obtained prior to the carotid intervention. For CEA, primary technical success implies the successful removal of the carotid plaque and closure of the artery, with or without a patch, with less than 30% residual stenosis in the absence of stroke, MI, and death. For CAS, the introduction and deployment of the EPD and carotid stent in the absence of stroke, MI, death, surgical conversion, and vascular obstruction constitute primary technical success [13]. Secondary endpoints of interest include procedure time, blood loss, blood transfusion, clamping and shunting time, fluoroscopy time, contrast load, recovery time, range and average number of days in an intensive care unit, and length of hospital stay.

All complications will be categorized as local vascular, local nonvascular, or systemic. Complications after carotid interventions will be reported in a systematic and standardized manner with a description of the degrees of severity. Although assigning a degree of severity to all complications arising from different treatment methods may be difficult, severity scales should be provided whenever possible to allow for the assessment and comparison of adverse events. The following severity scale has been modified from the reporting standards for lower extremity ischemia established by Rutherford et al [14]: *Mild* (level 1) refers to a complication that resolves spontaneously or with minimal intervention, does not increase the length of hospital stay, and does not cause permanent disability. *Moderate* (level 2) refers to the need for significant intervention, an extension of hospitalization beyond 24 hours, and, at most, minor permanent disability that does not interfere with normal daily activity. A *severe* complication (level 3) requires major surgical, endovascular, or medical intervention, may be associated with prolonged convalescence, is usually accompanied by prolonged or permanent disability, and may result in death. Prehospital discharge data related to stroke/death will be recorded, as applicable.

When obtaining patient consent, the patient will first be approached by a doctor who is a member of the treatment team at the same hospital in which the patient will be receiving treatment. Upon obtaining patient consent, we will record the patient name, patient signature, date of signature, and the name of the doctor who introduced the study to the patient, as well as his/her professional number and signature.

### Researcher Responsibilities, Institutions, and Sponsors

The principal investigators are committed to continuing the project over time while ensuring the accuracy of the information. Data will be collected from the routine diagnostic tests and specific treatment at each institution. There will be no project sponsor external to the universities.

The recruitment information will be available from the participating hospitals via an online document available to participants. In addition, the analytical procedure will include bimonthly meetings of the group of doctors responsible for the study, along with the respective principal investigators, to observe the progress of the project and evaluate the partial and total data, in addition to writing the manuscript.

### Statistical Analyses

Initial analyses will include sociodemographic characteristics of patients undergoing CAS, and those referred for CEA. The projected number of cases during the study period is approximately 800. Categorical variables will be compared and presented as percentages. Continuous variables will be compared using analysis of variance and will be presented as means with standard deviations. To identify patient characteristics that are independently associated with a referral for CAS versus CEA, no parsimonious multivariate logistic regression analysis of the probability of undergoing CAS will be performed. All analyses will be conducted using Microsoft Excel (Redmond, WA, USA) and Epi-Info (Atlanta, GA, USA). All significance tests will be performed at the .05 level.

## Results

The study is in the recruitment phase, and we are enrolling patients at 5 centers in Brazil. It is anticipated that 800 patients will be recruited to the study by the end of 2018. Analyses will focus on primary end points for patients that are allocated to each treatment group. During the per-procedural period, the occurrence of the primary end point components (stroke, MI, or death) for CAS and CEA will be analyzed for symptomatic or asymptomatic subjects. The median time from randomization to the procedure will be compared for CAS and CEA. Stenting with embolic protection of patients will be assigned to the CAS group. General or local anesthesia of patients will be assigned to the CEA group. The median duration of follow-up will be determined. During that time, the level or prevalence of selected risk factors will be analyzed to determine if they remained similar between the two treatment groups.

## Discussion

Clinical studies that evaluate carotid interventions, particularly those that compare different treatment modalities, may be difficult to interpret when differences in demographics, comorbid conditions, and perioperative risk factors are not identified and characterized [13,15].

The primary objective of the treatment for carotid stenosis is the reduction of risks related to stroke and death. Therefore, the primary outcome criteria for any carotid intervention include the prevention of the following: (1) all per-procedural strokes and death; (2) subsequent ipsilateral stroke; and (3) stroke or death that may result from primary or secondary treatment. There is agreement among health professionals that adequate training and experience of vascular surgeons is an important factor in maintaining the quality and outcomes of CAS or CEA, and this issue has been discussed in many reports following the results of European randomized controlled trials [16]. It has been suggested that CAS and CEA surgeons select an optimal strategy for each case, especially regarding protection methods, in accordance with preoperative risk evaluation. One of the major concerns associated with CAS is the potential for embolic infarction during the procedure. Among other causes of plaque components at the stenotic site, lipid core and plaque hemorrhaging are highly associated with increasing incidents of embolic infarction after CAS [17]. Multiple randomized trials have compared CAS with CEA, with varying results. Variability among the trials complicates efforts to make direct comparisons; thus, determining the best treatment strategy for symptomatic or asymptomatic patients is difficult.

One meta-analysis pooled data from 13 prospective, randomized, or controlled clinical trials that compared CEA with CAS [18]. With combined data from all trials, over 7000 patients were included, the majority of whom (79%) were symptomatic. The post operative risk of stroke and death over the subsequent 30 days was higher in the CAS group compared with the CEA group (odds ratio [OR] 1.57, *P*=.01) and was highest in symptomatic patients (OR 1.89, *P*=.01). EPDs used with CAS did not significantly reduce the 30-day per-procedural risk of stroke/death related to CEA, but they did decrease the 30-day risk of stroke/death compared to that associated with CAS without an EPD (2.7% vs 7.5%, OR 0.34, *P*<.01). In contrast, CAS was associated with a lower risk of cranial neuropathy (OR 0.06, *P*<.01) and a lower risk of post operative MI (OR 0.43, *P*<.01). Based on these findings, the authors advocated reserving CAS for revascularization in patients with anatomical conditions that make CEA difficult or place patients at higher risk for cranial nerve injury (such as restenosis after prior CEA), or in patients with concomitant significant carotid artery disease. The limitation of CAS to specific patient groups is outlined in both the Society of Vascular Surgery and European Society of Vascular Surgery guidelines, particularly for those at high risk for CEA and in, “high-volume centers with documented low per-procedural stroke and death rates or inside a randomized clinical trial” [19,20].

Some other important studies are underway. In response to the growing uncertainty regarding clinical management of asymptomatic patients with carotid artery disease, the National Institutes of Health is financing the CREST-2 trial. This multicenter, randomized study has two arms related to intervention: CEA versus best medical therapy (BMT) and CAS versus BMT. Patients can opt to enroll in either the CEA or CAS arm. Randomization, therefore, determines whether a patient undergoes an intervention or BMT, rather than the type of intervention (CEA or CAS). Patients allocated to CEA or CAS treatments will also receive the same BMT as those randomized to medical treatments. The study is expected to take approximately 10 years to complete randomization, and produce results for at least 4 years of follow-up [21].

The hospitals participating in this proposed study have a common characteristic: specifically, each is a large public university hospital involved in the training of new vascular surgeons within the most densely populated state of Brazil. This study, therefore, will represent a significant and ecologically valid sample for the treatment of carotid atherosclerosis in Brazil. Despite the existence of countless studies and international records pertaining to treatment for carotid artery disease, our study aims to obtain relevant data in the real-world of medical care in public university hospitals that are training future Brazilian vascular surgeons. Interactions between professionals who specialize in treating neurovascular diseases and vascular surgeons has become increasingly important in the ongoing search for the best approach to treating carotid disease, so the increased knowledge regarding the therapeutic results that are obtained in each region or country is of fundamental importance [22]. This knowledge will contribute very significantly to improving care for patients with carotid disease, in addition to qualifying the level of assistance provided in public university hospitals in the state of São Paulo, Brazil.

## Abbreviations

BMT: best medical therapy
CAS: carotid artery stenting
CEA: carotid endarterectomy
CREST: Carotid Revascularization Stent Trial
EPD: embolic protection device
ICSS: International Carotid Stenting Study
MI: myocardial infarction
OR: odds ratio
RHEUNI: Registry Project of Vascular Disease in the Public University Hospitals of São Paulo
